# Maternity care for trafficked women: Survivor experiences and clinicians’ perspectives in the United Kingdom’s National Health Service

**DOI:** 10.1371/journal.pone.0187856

**Published:** 2017-11-22

**Authors:** Debra Bick, Louise M. Howard, Sian Oram, Cathy Zimmerman

**Affiliations:** 1 Department of Women and Children’s Health, School of Life Course Sciences, King’s College London, St Thomas’ Hospital London, United Kingdom; 2 Section of Women’s Mental Health, Institute of Psychiatry and Neuroscience, David Goldberg Centre, King's College London, De Crespigny Park, London, United Kingdom; 3 Gender Violence and Health Centre, Department of Global Health and Development, London School of Hygiene & Tropical Medicine, London, United Kingdom; Leibniz Institute for Prvention Research and Epidemiology BIPS, GERMANY

## Abstract

**Background:**

Although trafficked women and adolescents are at risk of unprotected or forced sex, there is little research on maternity care among trafficking survivors. We explored health care needs, service use and challenges among women who became pregnant while in the trafficking situation in the United Kingdom (UK) and clinicians’ perspectives of maternity care for trafficked persons.

**Methods:**

Cross-sectional survey and qualitative interviews with trafficking survivors recruited from statutory and voluntary sector organisations in England and qualitative interviews with maternity clinicians and family doctors undertaken to offer further insight into experiences reported by these women.

**Findings:**

Twenty-eight (29%) of 98 women who took part in a large study of trafficking survivors reported one or more pregnancies while trafficked, whose data are reported here. Twelve (42.8%) of these women reported at least one termination of pregnancy while in the trafficking situation and 25 (89.3%) experienced some form of mental health disorder. Nineteen (67.9%) women experienced pre-trafficking physical abuse and 9 (32.%) sexual abuse. A quarter of women were trafficked for sexual exploitation, six for domestic servitude and two for manual labour. Survivors and clinicians described service challenges, including restrictions placed on women’s movements by traffickers, poor knowledge on how to access maternity care, poor understanding of healthcare entitlements and concerns about confidentiality. Maternity care clinicians recognised potential indicators of trafficking, but considered training would help them identify and respond to victims. Main limitations include that findings reflect women who had exited the trafficking situation, however as some had only recently exited the trafficking situation, difficulties with recall were likely to be low.

**Conclusions:**

More than one in four women became pregnant while trafficked, indicating that maternity services offer an important contact point for identification and care. Given the prevalence of sexual exploitation and abuse among trafficking survivors, clinicians should ensure antenatal care and screening for sexually transmitted infections can be readily accessed by women. Clinicians require specialised training alongside designated pathways and protocols with clear referral options to ensure confidential maternity care tailored to each woman’s needs.

## Introduction

Human trafficking is an international crime and globally recognised human rights violation defined as the recruitment and movement of people, using coercion, deception, and abuse of vulnerability, for the purpose of exploitation[1,2]. The International Labour Organization estimates that 11.4 million women and girls are in situations of forced labour as a result of human trafficking, of whom over four million are trafficked for forced sex work or sexually abused while exploited in other sectors[3]. Statistics from the United Kingdom (UK) on individuals identified as trafficked indicate that in 2015, 3,266 were referred to UK authorities, of whom 53% were female, most trafficked for sexual exploitation[4].

The trafficking of women and girls for forced sex has received growing attention over the past decade, with evidence regularly confirming women’s experience of violence, sexual abuse and poor health consequences, including unwanted pregnancies[5]. Research internationally has reported that levels of physical and sexual violence among women trafficked for sexual exploitation ranged from 33% in a Cambodian case file review[6] to 90% in a multi-country European study[7]. In one European study of 207 female survivors, 95% reported physical or sexual violence, with three-quarters reporting they had been physically hurt and 90% sexually assaulted[8]. Findings from a study from the Greater Mekong Sub-region (GMS) of 467 female survivors over the age of 15 found that 190 women of reproductive age had experienced sexual violence[9]. In the UK, 31% (n = 98) of survivors interviewed as part of the PROTECT study (Provider Responses, Treatment and Care for Trafficked People) experienced sexual violence while trafficked[10].

Female trafficking survivors frequently have multiple sexual and reproductive health care needs. A systematic review by Ottisova et al[5] found self-reported symptoms of sexually transmitted infections (STIs) in trafficked women who were sexually exploited ranged from 6% in a study from Israel to 66% in a cross sectional survey of sex workers in Thailand. Women also become pregnant while trafficked, as reported in the Greater Mekong region study, which showed that 35 (7.5%) of the 467 women interviewed became pregnant while in the trafficking situation[9]. Sex workers (*n* = 15) and brides/wives (*n* = 11) reported the greatest number of pregnancies and approximately one-third of pregnant respondents (34.3%, *n* = 12 of 35) had undergone an abortion. Not surprisingly, pregnancy is associated with abuse, as approximately three times as many women who experienced sexual violence were pregnant at the time of interview (7.4%, n = 14) compared with women who did not (2.5%, n = 7) [9].

Over half of 28 women survivors interviewed in one European study reported symptoms associated with STIs, including pelvic pain, pain or bleeding during intercourse, amenorrhea, heavy and irregular bleeding[11]. Lederer and Wetzel (2014)[12] in mixed methods study from the United States of health consequences and healthcare experiences of 107 women and girls trafficked for sexual exploitation found around two-thirds (67.3%) contracted some form of STI or other infection. Furthermore, just over half (55.2%) of the 67 respondents who reported a pregnancy had at least one abortion, with twenty women (29.9%) reporting multiple abortions. Trafficked women commonly come into contact with health services, although little is known of their experiences of these contacts. Lederer and Wetzel (2014)[12] found 88% of 98 survivors who answered a question on health service contacts saw a clinician during the trafficking situation, with data from PROTECT showing that 26 (19%) of 130 individuals (including 91 women) had accessed healthcare, most often family doctors (GPs)[13].

In this study we aimed to explore the experiences of women trafficked in the UK who became pregnant and had accessed maternity care provided by the National Health Service (NHS), a universal healthcare system funded by UK tax payers. We also explored the views of maternity care clinicians who had cared for trafficked individuals to offer further insight into women’s experiences. Study participants were recruited as part of the PROTECT study, details of which are published elsewhere[14].

## Methods

A cross-sectional survey of trafficked women, comprising a structured interview schedule and open-ended questions.To add insight to women’s experiences, we interviewed NHS clinicians who knew, or suspected women they were in contact with had been trafficked, to gain their perspectives on care offered and challenges facing trafficked women who are pregnant.

### Ethics statement

Ethical approval was provided by the National Research Ethics Service (NRES) Committee South East Coast–Kent (reference 13/LO/0099).

### Eligibility and recruitment

For the cross-sectional survey, we included trafficked women aged 18 years and over identified as a trafficked person by a statutory or voluntary agency; and those who had previously or were currently receiving assistance from one or more statutory or voluntary agencies. No restrictions were placed on exploitation type, time since exploitation, country of origin or language. Women were excluded if they were currently in the exploitation setting; too unwell or distressed to participate; or unable to provide informed consent.

#### Recruitment of women

Nine voluntary sector organisations offering government-funded post-trafficking support or authorised to refer potentially trafficked people for such support identified potential participants, with assistance from 10 healthcare providers and 10 social services departments. Caseworkers and healthcare staff approached a convenience sample of women eligible to participate with information about the study aims and procedures in their own language verbally and in writing, and liaised with the research team to arrange interviews.

#### Recruitment of NHS clinicians

We approached NHS clinicians who had treated individuals suspected or known to have been trafficked; or had adult or child “Safeguarding Lead” responsibilities in their organisation. In the UK, safeguarding refers to policies in organisations such as the NHS to protect vulnerable individuals’ health, wellbeing and human rights. Interviewees were identified through members of the PROTECT Research Advisory Group, the Project Steering Group, and by snowball sampling.

### Data collection

#### Trafficking survivors

Ninety-eight female trafficking survivors participated in the main PROTECT[14] survey, 28 of whom (29%) reported one or more pregnancies during the time they were trafficked whose data are reported in this paper. Interviews with trafficked women were conducted in participants’ current accommodation or agency premises. Travel and childcare expenses were reimbursed and women offered a £20 shopping voucher to thank them for their participation. Full details of recruitment procedures are provided elsewhere[14]. The interviews, which adhered to WHO guidance on ethical and safety recommendations for interviewing trafficked women[15] were conducted by trained researchers, supported by independent interpreters as required. Researchers emphasised the voluntary and confidential nature of the research, assured participants that the study was not related to immigration or policing processes, and explained participation (or non-participation) would not impact on the support from the referring agency or other organisations. Structured questions covered participants’ socio-demographic characteristics, trafficking experiences, and physical, mental, sexual and reproductive health (reported in full elsewhere[14]). Open-ended questions at the end of the interview explored participants’ access to and use of healthcare services during and since escaping the trafficking situation; this portion of the interview was digitally recorded with participants’ consent and transcribed verbatim. Questions were developed with assistance from the Project Advisory Group, piloted with the first four participants and revised accordingly.

#### Clinicians

We interviewed 29 clinicians in total as part of the PROTECT study[14], with data from 9 clinicians who provided maternity care presented here (three midwives, one obstetrician, one neonatal nurse and four family doctors). Face-to-face interviews with clinicians followed a topic guide and explored experiences of identifying, referring and caring for trafficked individuals, awareness of trafficking indicators, referral options, care pathways, barriers to care, and training needs. Topic guides were developed with assistance from the Project Advisory Group and revised following piloting.

Interviews lasted between 60 and 90 minutes and with the consent of participants were digitally recorded and transcribed verbatim.

#### Analysis

Descriptive statistics were calculated in Stata Version 11 (StataCorp LP, College Station, TX) including women’s socio-demographic characteristics, trafficking experiences, sexual, reproductive and mental health history. Framework analysis[16] was applied to qualitative data and analysis conducted in NVivo and MS Excel (99). Transcripts were read and reread, a priori themes refined, and new themes identified based on an initial sample of transcripts. A thematic framework based on the interview schedule and themes that emerged during data collection was initially applied to remaining transcripts and iteratively refined, followed by within and between-case analysis of themes. Interview extracts from trafficked women are attributed to participants by age group and type of trafficking. Pseudonyms are used to protect women.

### Results

#### Trafficked women’s characteristics

Women were between 18 and 41 years old and 18 (62.3%) were trafficked for sexual exploitation. Nine countries of origin were reported, although around three-quarters of women were from Albania (11, 39.3%) and Nigeria (9, 32.1%). Other countries of origin included Latvia, Romania, Slovakia, Pakistan, Moldavia, Ghana and Lithuania. Just over half of the women had exited the trafficking situation in the previous year. Nearly two-thirds (18, 64.3%) had one or more children, most of whom were living with them in the UK. During the trafficking situation, 9 women (32.1%) reported they were diagnosed with a sexually transmitted infection, 25 (89.3%) with some form of mental health disorder and 12 (42.8%) had undergone one or more terminations of pregnancy. Nineteen women (67.9%) had experienced pre-trafficking physical abuse and 9 (32.1) sexual abuse.

### Trafficked women’s experiences of accessing maternity care

The key themes that emerged in the qualitative analysis included: barriers to accessing maternity care; the importance of clinicians protecting patient confidentiality; positive and negative attitudes of clinicians; and the importance of continuity of care following birth.

#### Barriers to accessing maternity care

For many women, access to maternity care was frequently controlled by traffickers, although this was not the only barrier. Women reported delays to seeking care for fear of being charged a fee as a non UK resident, or because they did not know how to access NHS services. Women were also thwarted by needing to initially register with a family doctor who are ‘gatekeepers’ for NHS services, including maternity care.

Some women secured registration by providing a false address or getting a friend to register on their behalf. One woman explained how she sought registration but was refused because she lacked the correct documents:

When I was 4–5 months pregnant… I snuck out of the house and went to the local GP [family doctor] practice. When I arrived they told me I needed a passport and proof of address. I explained that I didn’t have this documentation and they turned me away (Augustina, late 20s, trafficked for sexual exploitation)

Barriers meant some women could only access maternity care late in pregnancy, which concerned their clinicians, as described by one woman who was approximately five months pregnant when she attended her first antenatal contact:

When I had my first visit at the hospital, the doctor she told me when she saw the baby, she told me that the baby seemed bigger than it should be. ‘You should have come earlier, actually, to see me’ (Sarah early 20s, trafficked for sexual exploitation)

Women described being asked to pay for maternity care even when this was not required, possibly as NHS staff were unaware of a woman’s status as a trafficked person or what this meant in terms of eligibility for care.

#### Importance of patient confidentiality

Women emphasised the importance of having their trafficking history treated confidentially by NHS clinicians. Lucy described how one doctor apologised for asking about her history, but needed to know as she had reported symptoms of a STI. When asked how she felt about this, Lucy explained:

It made me feel comfortable that everything is confidential–I wasn’t worried about everything being said, I was happy that if I’m gonna move from this area to another, it is OK for information to go to another doctor (Lucy, early 20s trafficked for sexual exploitation)

Some women did not experience the same level of confidentiality. Mercy, who spent 17 days on an antenatal ward where she received supportive, confidential care, was later admitted to another hospital to give birth. Here, her personal history was not treated as sensitively:

The doctors … were discussing my case but did it in a room with other people and by the door that led onto the corridor, so if I could hear other people could hear. In the (first) hospital, when they came to talk to me they would close the door so nobody else could come in. Even the police when they came to see me were in plain clothes..… nobody could find out about my situation (Mercy, late 30s, trafficked for domestic servitude).

Women also feared that if they disclosed their circumstances to clinicians, their traffickers would punish them for revealing the crime. This was particularly challenging when women were accompanied to antenatal appointments by those exploiting them. Isabel did not want to disclose any details about the person who accompanied her to an antenatal appointment:

If he [family doctor] asked me in the first place, I wouldn’t [reveal the true identity] because I would be scared. Even the guy that took me to the doctor–I just tell him they are like family friend (Isabel, early 20s, trafficked for sexual exploitation).

#### Women’s views of the maternity clinicians they encountered

Women’s views of the maternity clinicians they encountered were generally positive, however some reported negative attitudes. Carolina, who attended an NHS maternity unit because she thought her membranes had ruptured, explained her stigmatising encounter:

“Why are you here?” And they then wrote in the paper that you were trafficked, you was a prostitute. They not nice to you….and they was treating me exactly like, er, a prostitute.’ (Carolina, early 20s trafficked for sexual exploitation)

In contrast, Augustina who had a long labour praised the attitude of clinicians called to review her labour progress:

I had lots of doctors coming in and just to check; they were trying to discuss what to do and they were very good to me and I’m very very grateful to everybody that helped (Augustina, late 20s, trafficked for sexual exploitation)

Women also described the value of access to clinicians who could speak their language, as illustrated by Mercy, who was admitted to hospital after collapsing in the street:

They found all the nurses that worked in the hospital (who spoke the same language) and got one to come and talk to me to see how I was and to translate for me. They were really nice and friendly and they got me clothes, food and toiletries; one of the nurses even did my hair for me (Mercy, late 30s, trafficked for domestic servitude).

#### Planned continuity of care

Some women described the importance of scheduled health contacts following the birth, when clinicians could check their own and their infant’s health and offer support and advice on infant care. As Sarah explained:

They advised me to go to a children’s and new mother’s group, to get with other mothers, there are a lot of things I can do. They weighed him and checked his eyes and advised me about breastfeeding (Sarah, early 20s, trafficked for sexual exploitation)

These contacts offered particular reassurance to women concerned about their infant’s health, as Rachel noted:

I’ve been worr[ied about] hepatitis, I say, like if my baby can [get] hepatitis from me. And they try to explain me many many times, like after the delivery, they came and checked the baby. And after three or four injections she is 100% protect[ed] from hepatitis (Rachel, early 30s trafficked for sexual exploitation).

An ongoing concern was poor continuity of care from family doctors. Julia, who suffered anxiety and sleep disorders, reported how she had to see a different family doctor each time she had an appointment even though they were based in the same practice:

The one I know before (family doctor) that I was used to, maybe then next time I go again I sit there and I see another face and I have to get use to another face again. As soon as you meet a new doctor, they give you a new prescription. (Julia, early 30s trafficked for sexual exploitation)

In the UK, trafficking survivors maybe relocated or ‘dispersed’ by immigration authorities to different areas of the country[17]. Women who were relocated were obliged to re-register with a new family doctor, often fearing more barriers to access the care they needed. As Tanya explained:

Because I just moved one month ago, and actually I need to go to my appointments for my liver and hepatitis to check everything. And to transfer me here (to another part of the country), it takes three to four months [to sort paperwork needed to register with a new family doctor] they say to me (Tanya, late 20s, trafficked for sexual exploitation)

### Interviews with maternity care clinicians

To provide further insight into experiences of trafficked women, data from nine clinicians who provided maternity care are presented. Findings included: training needs; awareness of indicators of trafficking; barriers and resource constraints; and continuity of care.

#### Training needs

All clinicians received mandatory training on safeguarding and care of vulnerable patients, but none had received specific training on identifying, assessing or caring for potential victims of trafficking. As one clinician explained, they tried to glean information through their work experiences:

We don’t get a specific class that’s about trafficking. Much of what I’ve learned has been informal sort of on the job osmosis (clinician 1)

Another clinician referred to awareness of indicators of trafficking, but specific training:

‘Would be helpful to know what other things may prompt us to think about it in terms of medical symptoms and signs of people with depression or PTSD, which they’re not disclosing and how to start the conversation (clinician 6).

Clinicians suggested that issues related to trafficking could be included within their mandatory training and were not averse to this potential. The importance of knowing about women’s human rights and entitlements to care was raised by several interviewees, some of whom referred to their poor knowledge of referral processes, including how and when to involve the police, statutory and voluntary agencies.

#### Awareness of indicators of trafficking

Clinicians described several indicators of trafficking based on their experiences. These included late booking for maternity care, a woman unable to speak or read English or with limited knowledge of the area where she lived. As one clinician described:

They’d appear at you know more than 20 weeks pregnant—it could be 36 weeks pregnant and what’s particularly disturbing is they don’t know where they live and they have no English language skills’ (clinician 3)

Another potential indicator was if the individual woman or the person who accompanied her were reluctant to accept the offer of a case worker to help with English language translation:

‘..when we ask them to see the doctor or nurse by themselves with the case worker, there’s sometimes a barrier put up, or the person with them is a bit ‘anti’ this offer, or they look scared’ (clinician 8)

In the case of younger women, being accompanied to appointment by adults who referred to themselves as an “aunty” or “uncle” raised concerns. As one clinician explained:

We became quite suspicious because both the aunty and a man were with her, supposedly it could be an uncle. And when we dig further, we actually found out that this ‘aunty’ brought over this girl from Pakistan. (clinician 2)

The same clinician advised that if there were suspicions someone had been trafficked, they should seek further information, for example clarification about a woman’s next of kin, but added NHS staff may be fearful of being perceived as ‘judgmental’.

#### Healthcare barriers and resource constraints

Problems registering with a family doctor were major barriers to women accessing maternity care, sometimes due to poor awareness among NHS staff (including administrative staff) about women’s entitlements to NHS care:

They (women) probably have to go around to different [family doctor]practices until they find one that knows that they should be providing care for them. I do think there is an issue in the NHS in general, a lack of understanding about who is entitled to what. I think that is a huge issue (clinician 4)

One clinician described how one trafficked woman had a significant wait for psychiatric care because she was unable to register with a family doctor to obtain the necessary referral. NHS resource constraints were also problematic. Even when women were in the ‘system’, barriers presented if an interpreter was needed but clinicians had to negotiate which NHS budget their costs would be paid from.

#### Continuity of care

All clinicians highlighted the importance of continuity of care, but if a woman was suddenly relocated to another part of the UK by immigration authorities, their ability to support women was limited.

[She] was pregnant with twins and we realised on close questioning was sleeping in a church and when we tried to sort her out, the Home Office sort of whisked her away to another part of the country (clinician 3).

One individual discussed the responsibility clinicians felt to make sure women had a safe place to live and support necessary to meet their needs in these vulnerable situations, but there was little they could do:

I do worry about ongoing care of these women or girls and their children and babies once they’re born. They’ve got no one to teach them parenting…They’re moved away and don’t even know where they are or where they are going to. (Clinician 3).

The relocation of women generated significant administrative work for clinicians tasked with co-ordinating the handover of care:

That was let’s say, a day’s work of phone calls, using the internet. You know the area of country, got through to the GP [family doctor], got through to the key worker, and I think we had an interpreter there, that worked well…..because when people move around the country it can be extraordinarily difficult. (clinician 2)

Clinicians rarely had relevant, timely information to plan appropriate handover and lacked pathways and protocols to promote seamless transfer from one NHS organisation to another.

## Discussion

This is the first study to specifically explore the experiences of women who accessed maternity care during the trafficking situation. Findings indicate that maternity services can play an key role in the identification, support and appropriate referral of trafficked women and girls. Our findings that one in four women became pregnant when trafficked, combined with findings that maternity clinicians were more likely than any other clinical group to have had contact with a victim of trafficking[18] highlights the importance of ensuring maternity professionals are prepared to identify and care for trafficked women.

Women and clinicians described various barriers to securing access to maternity services. In some cases, surveillance or restrictions by traffickers prevented women from seeking care and in other cases, women themselves felt inhibited because they did not have the correct documents, did not know how to access NHS services or feared they could not afford care if they had to pay. Our findings are similar to those of Konstantopoulos et al (2013)[19] who undertook a comparative analysis of general healthcare use among women and girls trafficked for sexual exploitation in eight global cities, and the systematic review of experiences of maternity care among immigrant and non-immigrant women by Small et al (2014).[20] Both studies found that unfamiliarity with care systems impacted negatively on women’s experiences. To prevent adverse maternal and fetal outcomes, trafficked women who become pregnant require carefully planned care given their substantial risk of co-morbidity, including high rates of STIs, mental health problems, previous experiences of physical and sexual abuse and complex social problems.[21]

The women who accessed maternity care often remained concerned about confidentiality and expressed their relief that clinicians were cautious about keeping their information confidential. Breaches of confidentiality were especially worrisome for women who feared their details would be provided to immigration authorities or that their traffickers would punish them for sharing information about their crime. The importance of confidentiality is highlighted in WHO guidance on responding to domestic violence in clinical settings for women and young girls[22] and the International Organization for Migration guidance for healthcare providers[23]. Similar guidance for victims of domestic abuse from the UK and other countries recognize fear of disclosure for reasons similar to those identified in our study[24,25,26].

Women generally expressed satisfaction with their maternity care, reporting involvement in decision making and care respectful of their needs and circumstances. Nevertheless, some encountered negative attitudes. Concepts associated with ‘respectful maternity care’ as a universal human right are even more fundamental for women who have been trafficked. Global policy for maternity care reflects women’s dignity, equality and safety as core principles [27,28,29,30]. The clinicians we interviewed considered that mandatory training for healthcare staff should include trafficking, with respectful care and human rights core to training content. Our PROTECT survey of nearly 800 clinicians similarly identified high levels of support for training, with over three quarters of respondents reporting an interest in future training on trafficking issues[18].

In common with immigrant women’s experiences of maternity care in high income country settings[20], trafficked women decribed postnatal contacts as important to support breastfeeding and monitor infant growth and development. Postnatal care is a ‘missed opportunity’ generally to support maternal and infant health[31]. A recent cross sectional analysis of length of stay data from healthcare facilities in 92 countries found substantial proportions of women had postnatal inpatient durations too short to enable them to benefit from interventions available[32]. Mother-infant interaction is a key mediator of the relationship between maternal mental health difficulties and adverse child mental health outcomes, particularly in conditions of poverty and chronic mental health difficulties[33]. To prevent inter-generational transmission of vulnerability, and adverse mental and physical child health outcomes, women should be offered ongoing targeted support for their mental and physical health and parenting of infants conceived in the traumatic setting of trafficking. Where post-discharge care is available internationally, services need to assess and support women, as their histories of complex traumas and high levels of psychological symptoms are likely to impact on their ability to interact optimally with their infants.

An issue for continuity of care, particularly following the birth, was the potential for women to be relocated to another part of the country. Resources and systems to refer women who are relocated to the appropriate government and non-government agencies need to be reviewed, particularly in areas with higher numbers of trafficked individuals. Recent UK guidance on healthcare needs and pregnancy dispersal for those seeking asylum recognises the risks to maternal health, with recommendations that caseworkers try to avoid relocation of pregnant and postnatal women because of the impact on their ongoing care[34]. If relocation is unavoidable each woman’s case should be treated and managed to reflect their individual circumstances.

UK law, like legislation in a number of other countries, ensures that individuals who are identified as a potential victim of trafficking are exempt from healthcare charges[35] but not all survivors wish to be referred to the authorities e.g., for fear of retribution by traffickers[36]. Additionally, many countries do not have rights to free health care, including for trafficked individuals who could be viewed as a specific sub-group of undocumented migrants[37]. In countries that have provisions to grant access to care for trafficked persons, it will be important for clinicians to understand trafficked people’s rights and entitlements. As well as encouraging greater use of maternity services, this knowledge may also promote earlier use of maternity and other healthcare and greater continuity of care [25,38].

The study had several important limitations. As we opted not to include women still in the trafficking situation due to ethical and safety concerns, our findings reflect women who had exited the trafficking situation. Consequently, we cannot confirm that they reflect the circumstances of the wider population of women who remain unidentified. Generalisability of findings from studies of trafficked people is likely to be limited, as individuals may not access support. If those who do seek support have more extreme healthcare needs, studies may overestimate health risks. Conversely, if individuals who are less harmed are more likely to access support, findings could underestimate risk[39]. While seven women were pregnant at the time of interview, much of the information collated was retrospective and as such, recall difficulties–and the potential for bias—cannot be ruled out. However as some women had only recently exited the trafficking situation prior to being interviewed, we anticipate this to be minimal. Additionally, while our findings are particularly relevant for high income countries with universal healthcare, information about women’s experiences are likely to be applicable to other settings, as barriers to maternity care, importance of continuity of care, respectful care and training needs of clinicians are unlikely to be unique to UK maternity services. Furthermore for a study on human trafficking our study sample was relatively large, and as we interviewed women with experiences across various circumstances of exploitation, we believe that findings provide substantial insights for future maternity care services.

## Conclusion

Front line maternity services are important to the identification, support and referral of trafficked women and girls. Women described mostly positive experiences of contacts with maternity care clinicians, who respected their confidentiality and provided the care women and their infants needed. Continuity of care was particularly valued. Barriers to accessing maternity care, particularly in early pregnancy, should be addressed given the prevalence of sexual exploitation and abuse among trafficking survivors. Evidence-informed protocols and pathways should be available for all staff, particularly to support ongoing care of women and their infants who may be relocated, and enable those who provide maternity care, including postnatal care, to access the training they need.

## Supporting information

S1 Appendix Developing the codes. (DOCX)

S2 Appendix Framework analysis example. (DOCX)
