# Supplementary material for: Maternity care for trafficked women: Survivor experiences and clinicians’ perspectives in the United Kingdom’s National Health Service
Source: PLoS One. 2017 Nov 22;12(11):e0187856. doi: 10.1371/journal.pone.0187856 (PMC5699814; doi:10.1371/journal.pone.0187856)
Supplement: S1 Appendix — (DOCX) [file pone.0187856.s001.docx]

**S1 Appendix: Developing the codes**

| **CODE** | **DESCRIPTION** |
| --- | --- |
| **Access to GP services** | |
| Trying to access GP registration | *Not knowing how to access the NHS system; prevented referral to other health services; delayed discharge from inpatient care* |
| Women should not be ‘dropped’ by GP practices | *Having to see different doctors; impact on postnatal care; GPs lack of awareness of woman’s trafficking circumstances* |
| Uncertainty about need to pay for healthcare | *Delay in registering for GP care; unsure if hospital maternity care would need to be paid for; delay in seeking pregnancy care* |
| Stress of trying to access GP care | *Not having relevant papers; trafficker blocking women from seeking GP contact; woman having to take ‘friend’; concern about need for ongoing and future healthcare needs;fear of not getting healthcare when needed* |
| **Maintaining confidentiality** | |
| NHS staff awareness of trafficking | *Not wanting to repeat trafficking history, embarrassment, fear of being returned to trafficker* |
| Trust in NHS staff | *Protection of details, confidence in care, details not being passed on without woman’s permission* |
| Need to reassure other trafficked women | *Trafficked women need to know confidentiality about their history will be protected by NHS clinicians* |
