# Supplementary material for: Maternity care for trafficked women: Survivor experiences and clinicians’ perspectives in the United Kingdom’s National Health Service
Source: PLoS One. 2017 Nov 22;12(11):e0187856. doi: 10.1371/journal.pone.0187856 (PMC5699814; doi:10.1371/journal.pone.0187856)
Supplement: S2 Appendix — (DOCX) [file pone.0187856.s002.docx]

**S2: Framework analysis example: Access to family doctor. Quotes underlined used in main text**

|  | **Barriers to GP access** | **GP practice registration process** | **GP being aware of trafficking history** | **Importance of GP care during and after pregnancy** |
| --- | --- | --- | --- | --- |
| **Linda** (early 20s) | I didn’t have the correct paperwork – I didn’t know what to do | My friend, she tried to help me | It was not easy situation for me. Erm yeah, they believed me, they tried to help me as well but I’m little upset when they cancel me | Because the hospital says to me ‘your GP cancelled you. How can we discharge you as you don’t have a GP. And I been in hospital 10 days! |
| **Sarah** (early 20s) | I needed to see GP because I was pregnant, but the GP wouldn’t register me without any papers from the Home Office | The registration with the GP was quite stressful. That was very difficult as I didn’t have papers from the Home office. | I wouldn’t feel comfortable if they had asked me because I feel really embarrassed and ashamed. | When I had my first visit at the hospital, the doctor she told me when she saw the baby, she told me that the baby seemed bigger than it should be. ‘You should have come earlier, actually, to see me’ |
| **Isabel** (early 20s) | What I don’t like about the GP in general is the fact that they are always in a rush. They make you wait sometimes a week for an appointment | I was taken to the GP to register by my trafficker. And he was there with me. So I wasn’t really comfortable to tell him stuff what I would need to tell | In a way, I feel like my first GP….I feel like they should have seen the signs something wasn’t right | It’s just that I don’t think (health needs) are being taken seriously |
